# Investigating the Irish brain drain: factors influencing migration intentions among medical students

**DOI:** 10.1186/1753-6561-9-S7-A12

**Published:** 2015-10-27

**Authors:** Kevin Kitt, Pishoy Gouda, David S Evans, Deirdre Goggin, Deirdre McGrath, Jason Last, Martina Hennessy, Richard Arnett, Siun O'Flynn, Fidelma Dunne, Diarmuid O'Donovan

**Affiliations:** 1 National University of Ireland, Galway, Ireland; 2 Senior Research Officer, Department of Public Health, Galway, Ireland; 3 Assistant Staff Officer, Department of Public Health, Galway, Ireland; 4 Director of Education, Graduate Entry Medical School, University of Limerick, Limerick, Ireland; 5 Associate Dean for Programmes and Educational Innovation, UCD School of Medicine & Medical Science, Dublin, Ireland; 6 Associate Professor, Education Division, School of Medicine, Trinity College Dublin, Dublin, Ireland; 7 Associate Director of Quality Enhancement Office, Royal College of Surgeons in Ireland, Dublin, Ireland; 8 Head of Medical Education, University College Cork, Cork, Ireland; 9 Personal Professor/Consultant Endocrinologist, College of Medicine Nursing and Health Sciences (CMNHS), Galway, Ireland; 10 Senior Lecturer in Social and Preventive Medicine, Galway, Ireland

## Background

Ireland has the highest level of medical emigration in Europe with an increasing demand for physicians worldwide [1,2]. This has received considerable public and political interest. However, few studies have described the migration intentions of medical students at the undergraduate level [3]. Our study aimed to describe the migration intentions of Irish medical students by nationality and identify unique factors, “push factors”, that influence their decisions.

## Methods

Cross-sectional online survey of medical students in Ireland. Pearson's Chi square was utilised to determine the significance of differences in migration intentions and factors influencing migration. Free text data was thematically analysed.

## Results

Of 2273 respondents, 67% were Irish, 5.3% were from other EU countries and 27.8% were from Non-EU countries. 88% of Irish students identified that they were definitely or contemplating going abroad following graduation/intern year, compared to 80% of Non-EU students and 88% of other EU students (P<0.001). Training and career aspects, personal development and financial reasons were identified as key “push” factors influencing migration intentions.

## Conclusions

It would be expected that a large proportion of EU and non-EU students would migrate from Ireland following training. However, it is alarming that the intention to migrate is significantly greater among Irish students than non-EU students. As eight out of ten of all students are considering migration, initiatives to support retention should target all nationalities and their unique “push” factors that have been identified.

## References

[B1] García-Pérez MA Amaya C Otero Á Physicians' migration in Europe: an overview of the current situation BMC Health Serv Res 2007 7 201 18070353 10.1186/1472-6963-7-201 PMC2248190

[B2] Scheffler R Liu J Kinfu Y Dal MR Poz Forecasting the global shortage of physicians: an economic- and needs-based approach Bulletin of the World Health Organization 2008 86 516 523 18670663 10.2471/BLT.07.046474 PMC2647492

[B3] Burke C Survey of Final Year Medical Students 2012: will we go or will we stay? 2012 1 46

